# Supplementary material for: Early warning of trends in commercial wildlife trade through novel machine-learning analysis of patent filing
Source: Nat Commun. 2024 Aug 1;15:6379. doi: 10.1038/s41467-024-49688-x (PMC11294592; doi:10.1038/s41467-024-49688-x)
Supplement: Supplementary file 1 — Supplementary Information [file 41467_2024_49688_MOESM1_ESM.pdf]

Supplementary materials

Appendix 1: Summary statistics for patent filing

- **Table S1**

Appendix 2: All changepoint and event timelines

- **Figures S1-S6**

Appendix 3: All manual topic model graphs

- **Figures S7-S12**

Appendix 4: Manual topic keywords

- **Table S2**

Appendix 1: Summary statistics for patent filing

Table S1: Patent filing trends for six taxa between 1970 and 2020, scraped from the Google Patents database

Taxa	Median monthly patents 01-1988 to 01-1989	Median monthly patent 06-2019 to 06-2020	Total patents 01-1970 to 06-2020	First patent filed	Maximum patents per month	Month with maximum patents	Changepoint month	Mean change % 1988-2020
Bear	0	5	1718	01-1977	34	2015-12	1992-03	115
Caterpillar fungus	0	46	14754	08-1978	485	2015-12	1992-08	143
Horseshoe crab	0	2	1209	05-1970	13	2017-09	1987-08	102
Pangolin	0	8	6907	03-1984	150	2013-12	1995-08	130
Rhinoceros	0	1	526	07-1988	53	2013-01	2008-03	149
Sturgeon	0	8	2194	02-1971	117	2016-04	2003-04	129
Global (all patenting activity)	127,233	406,270	118,393,322	-	688,747	2017-12	-	104
Patents from China	0	327,456		-	559,551	2019-12	-	-

Appendix 2: Changepoint and event timelines

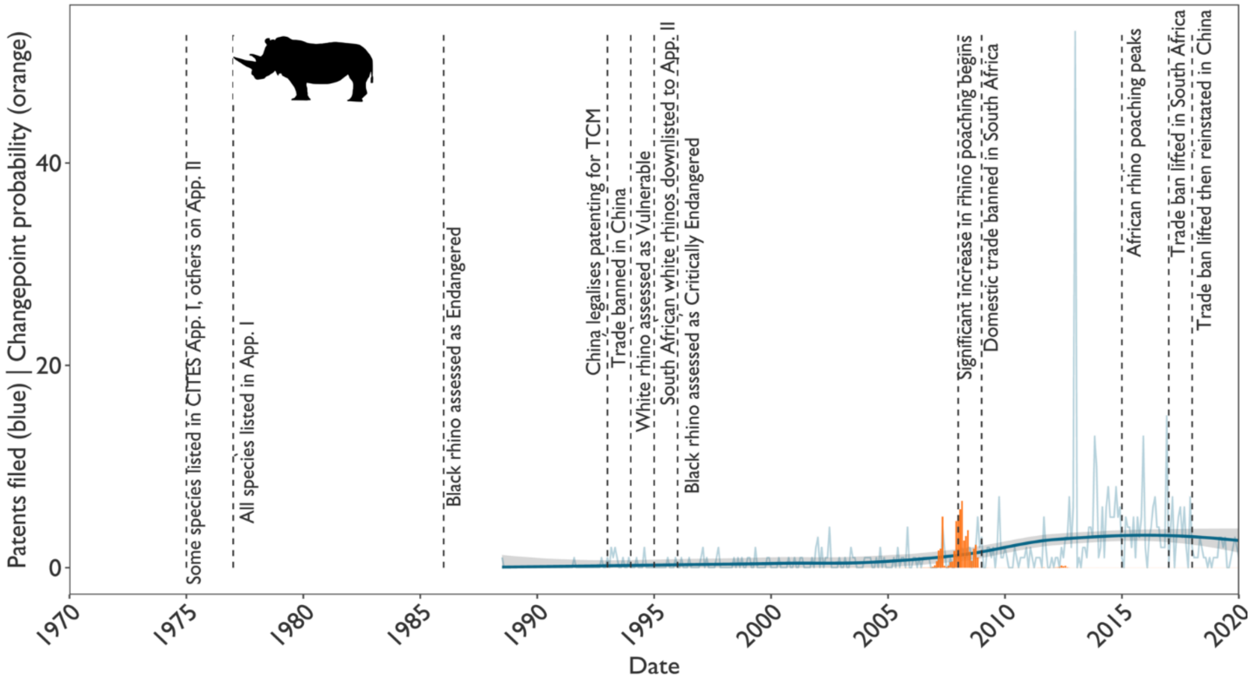

Figure S1. Changepoint and trends in patent filing for rhino-related innovations between 1970 and 2020, overlaid with a timeline of key rhino trade and conservation events. Silhouette: www.phylopic.org/images/fdfaac80-2382-4d80-888b-bca2ca3ca2e7/ceratotherium-simum-simum (Public Domain)

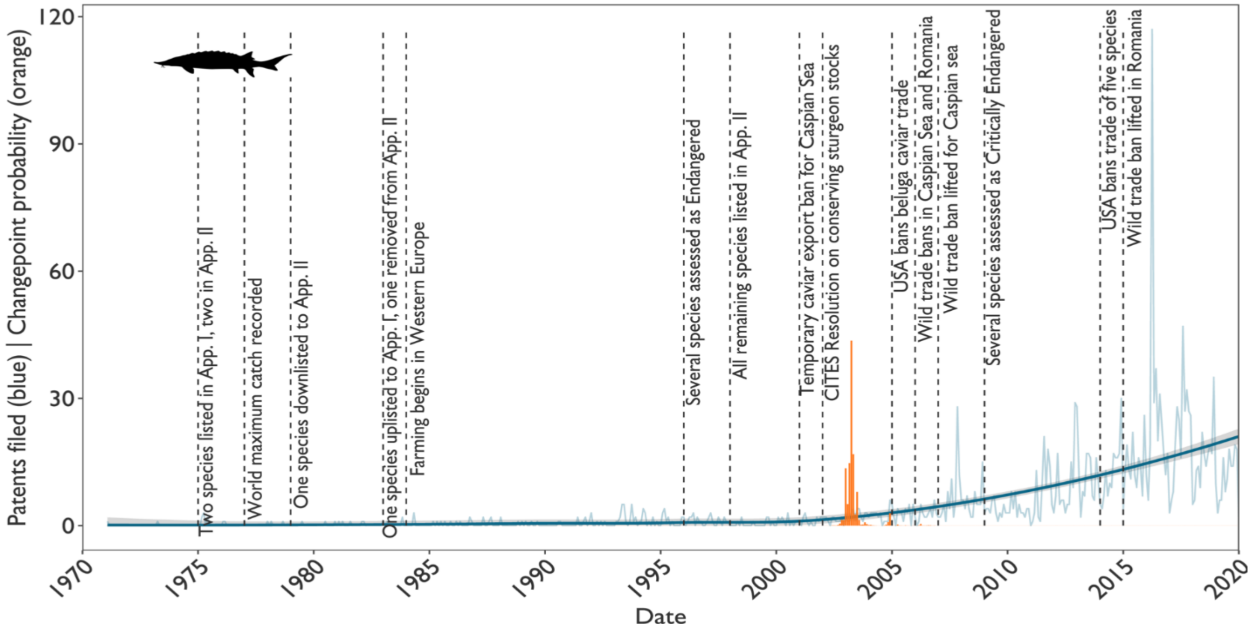

Figure S2. Changepoint and trends in patent filing for sturgeon-related innovations between 1970 and 2020, overlaid with a timeline of key sturgeon trade and conservation events. Silhouette: www.phylopic.org/images/5ce55e7c-827d-4049-b128-ae0bc8fb2981/acipenser-oxyrhynchus (Public Domain)

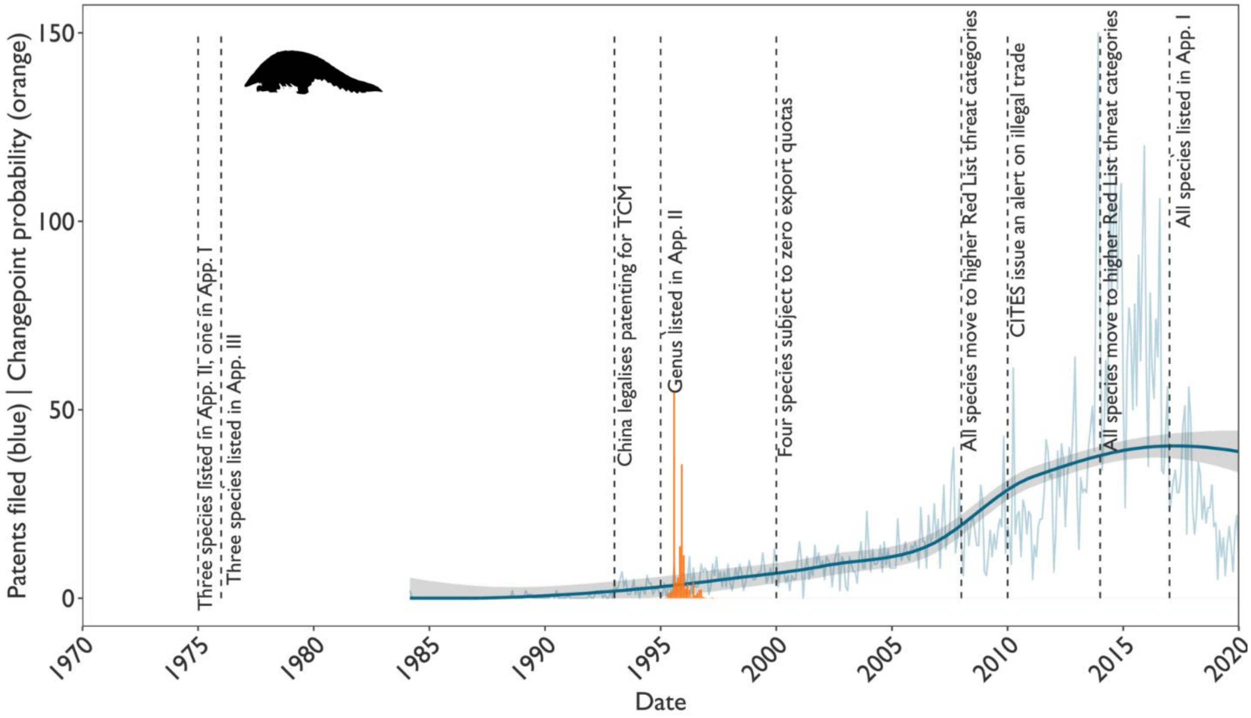

Figure S3. Changepoint and trends in patent filing for pangolin-related innovations between 1970 and 2020, overlaid with a timeline of key pangolin trade and conservation events. Silhouette: www.phylopic.org/images/f628f6fe-6e2d-4173-a20f-265dc7d2d04f/manis-culionensis (Public Domain)

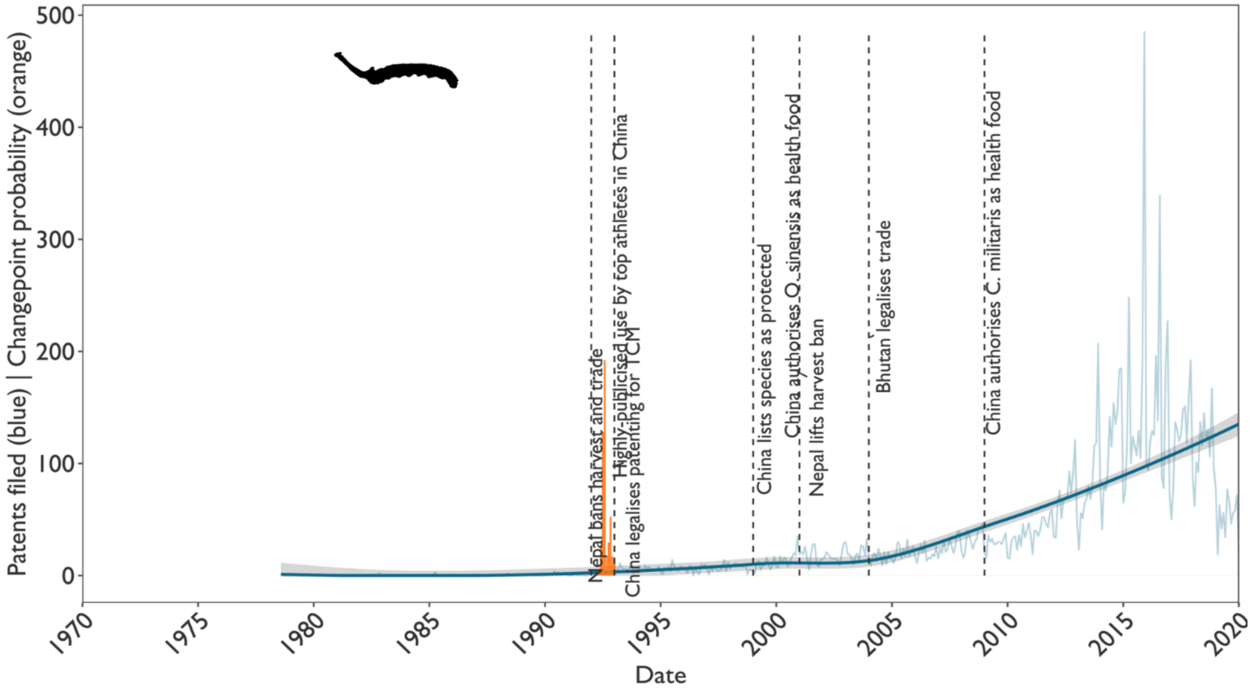

Figure S4. Changepoint and trends in patent filing for caterpillar fungus-related innovations between 1970 and 2020, overlaid with a timeline of key caterpillar fungus trade and conservation events. Silhouette image adapted from original photograph at https://commons.wikimedia.org/wiki/File:Cordyceps_Sinensis.jpg uploaded by William Rafti. The

original image is licensed under the Creative Commons Attribution 2.5 Generic license: https://en.wikipedia.org/wiki/en:Creative_Commons

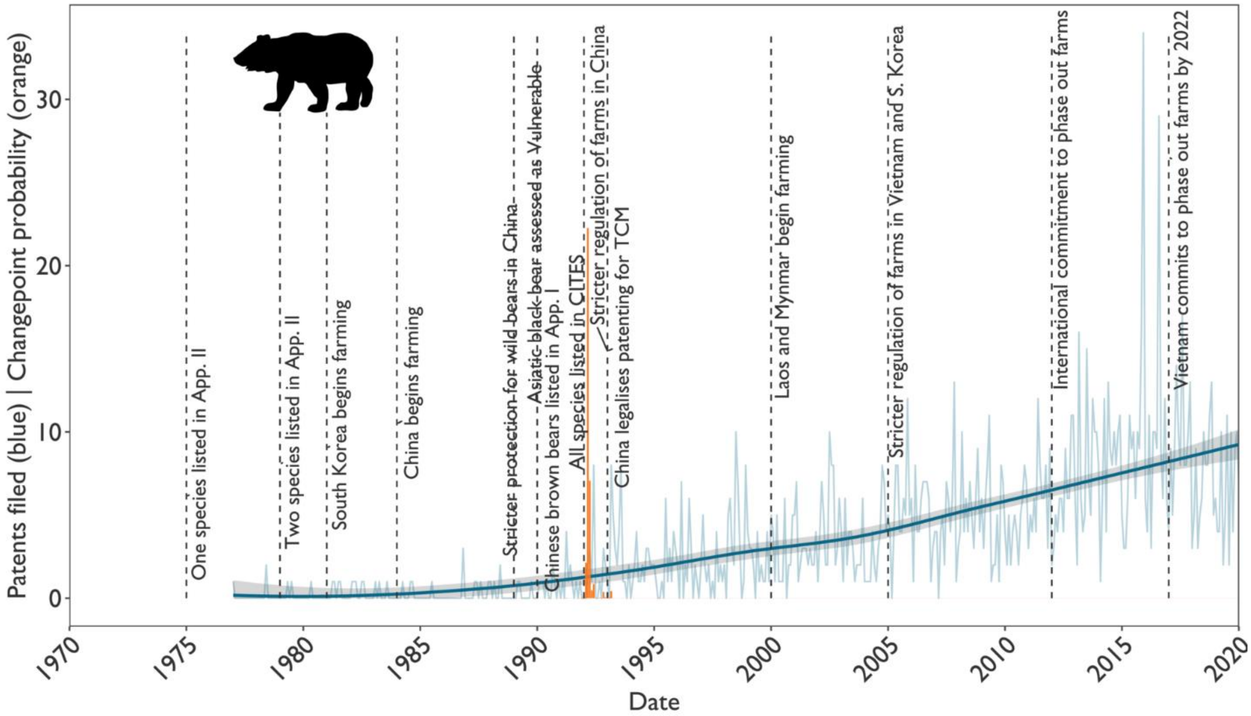

Figure S5. Changepoint and trends in patent filing for bear-related innovations between 1970 and 2020, overlaid with a timeline of key bear trade and conservation events. Silhouette: www.phylopic.org/images/51f6ff66-3344-4373-b6b0-e8beeb7fee33/ursus-thibetanus (Public Domain)

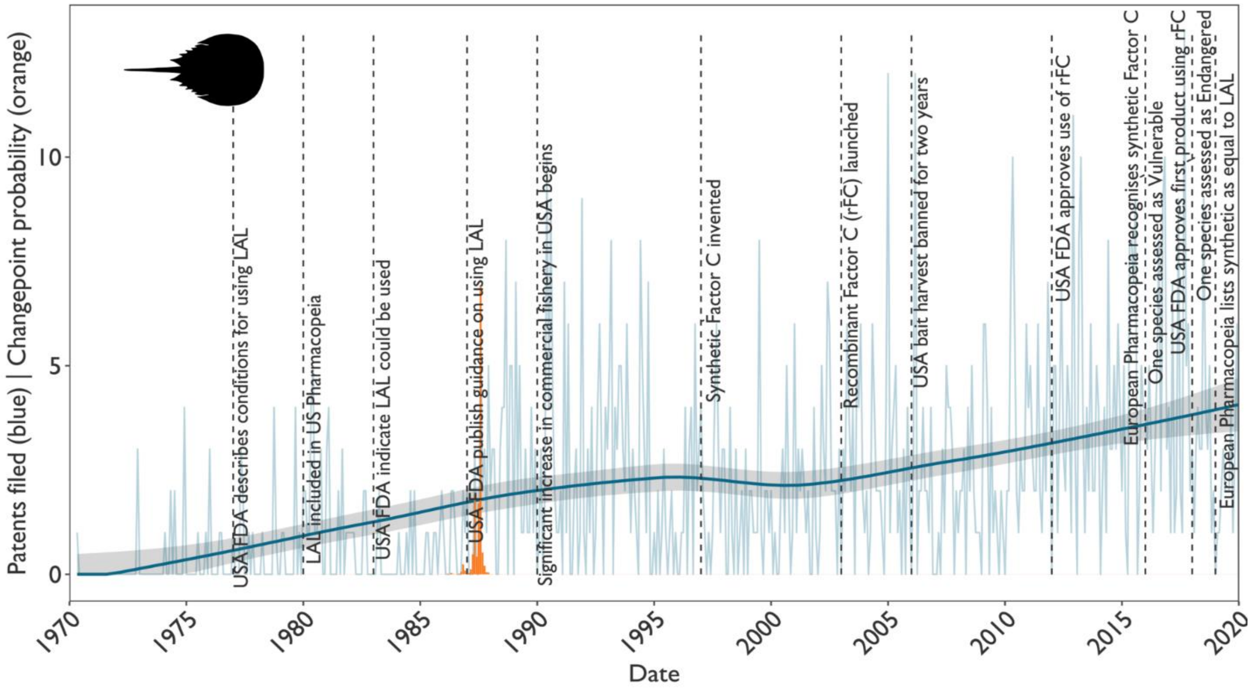

Figure S6. Changepoint and trends in patent filing for horseshoe crab-related innovations between 1970 and 2020, overlaid with a timeline of key horseshoe crab trade and conservation events. Silhouette:

www.phylopic.org/images/9a9a0cd0-2fb0-4ab7-b0bc-5984d11e5c13/limulus-polyphemus (Public Domain)

Appendix 3: Manual topic model graphs

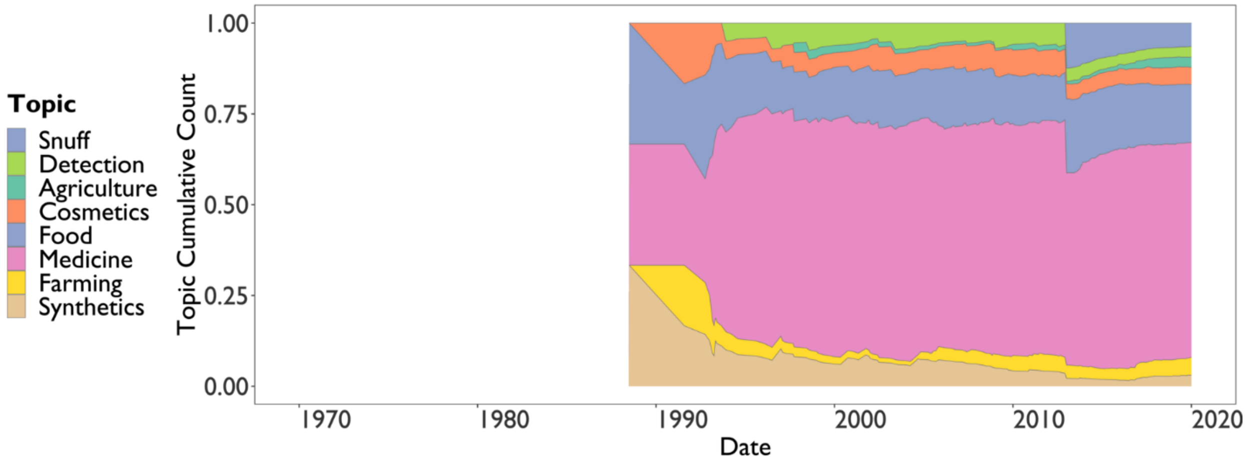

Figure S7. Manual topic model graph showing trends in topics for rhino-related patents between 1970 and 2020

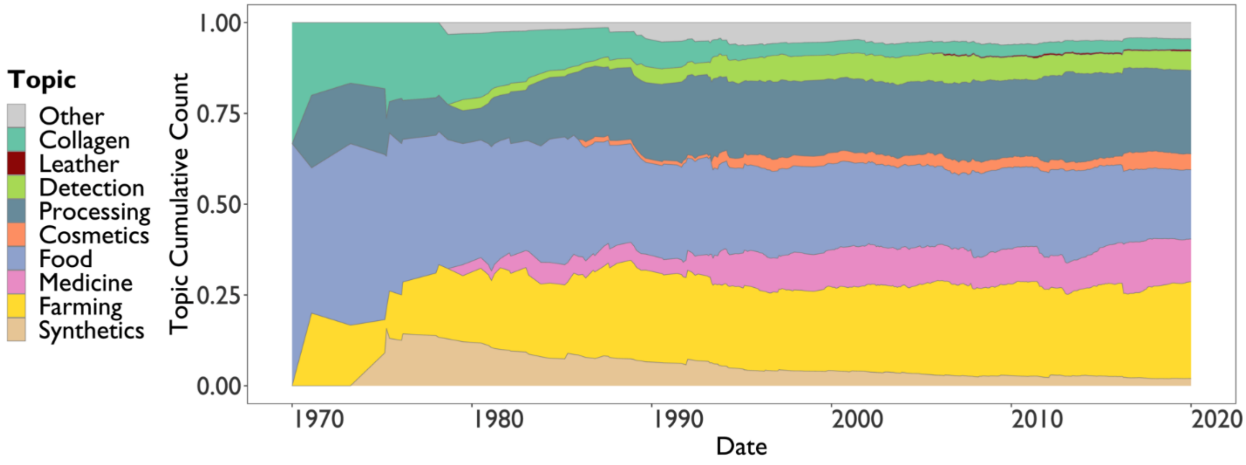

Figure S8 Manual topic model graph showing trends in topics for sturgeon-related patents between 1970 and 2020

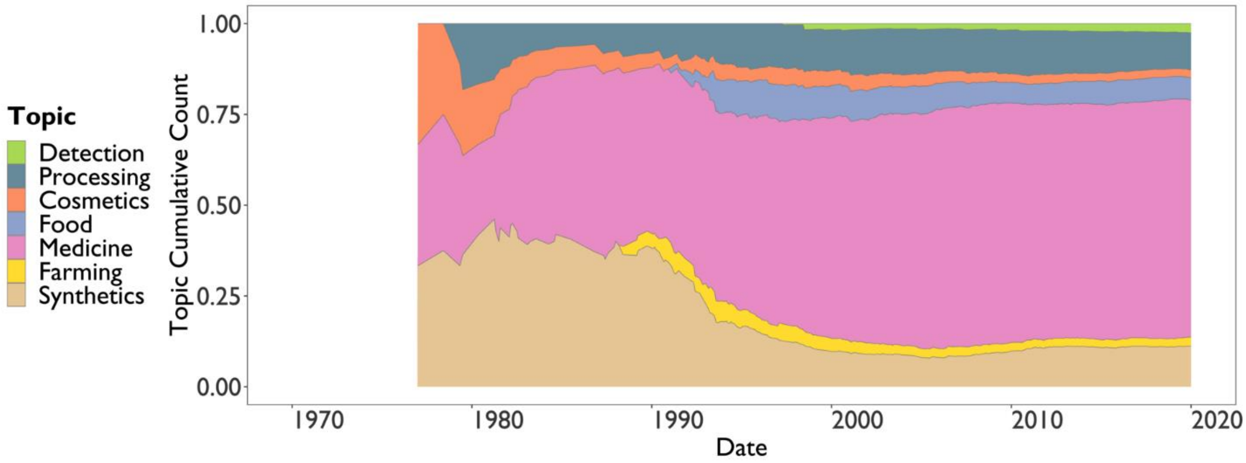

Figure S9 Manual topic model graph showing trends in topics for pangolin-related patents between 1970 and 2020

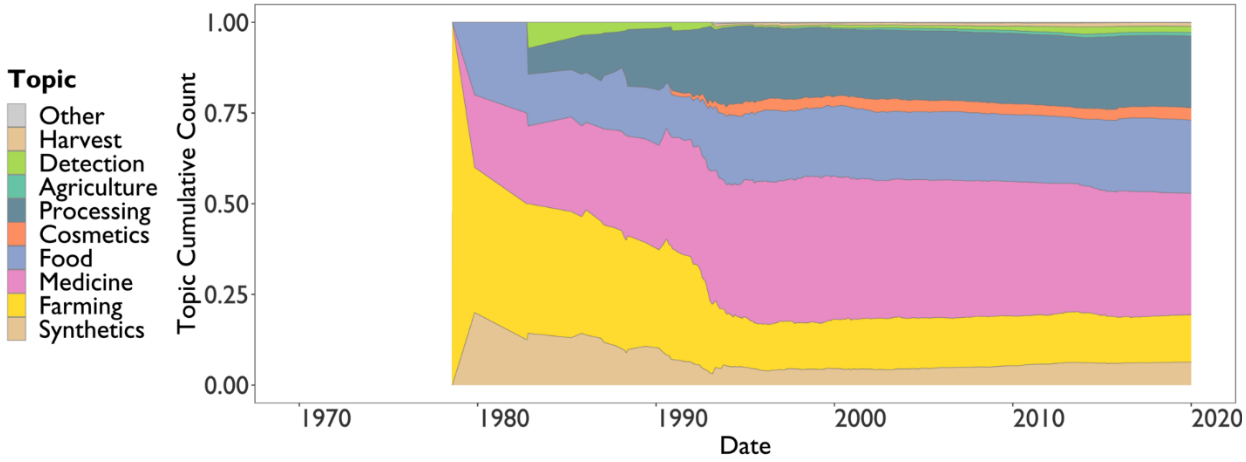

Figure S10 Manual topic model graph showing trends in topics for caterpillar fungus-related patents between 1970 and 2020

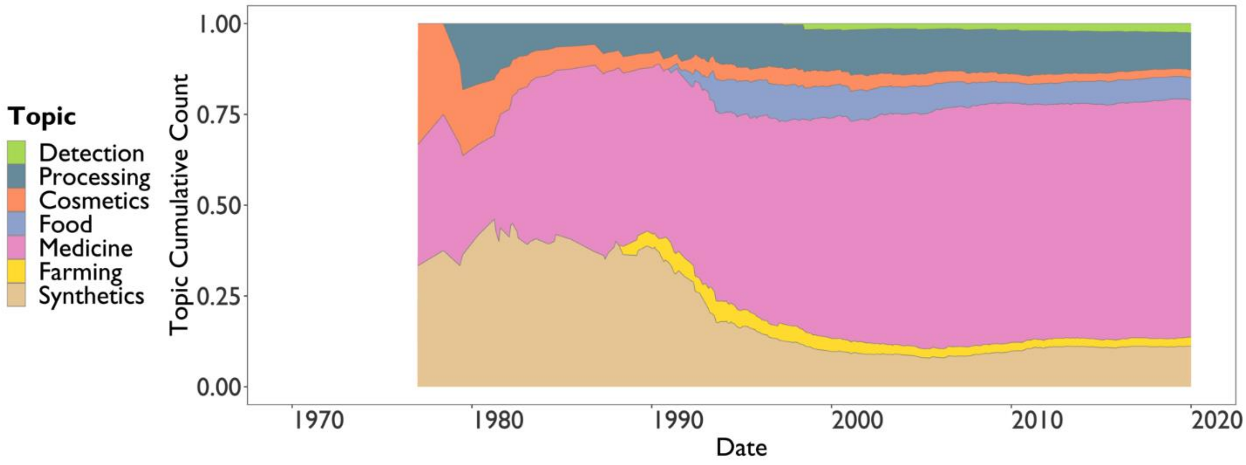

Figure S11 Manual topic model graph showing trends in topics for bear-related patents between 1970 and 2020

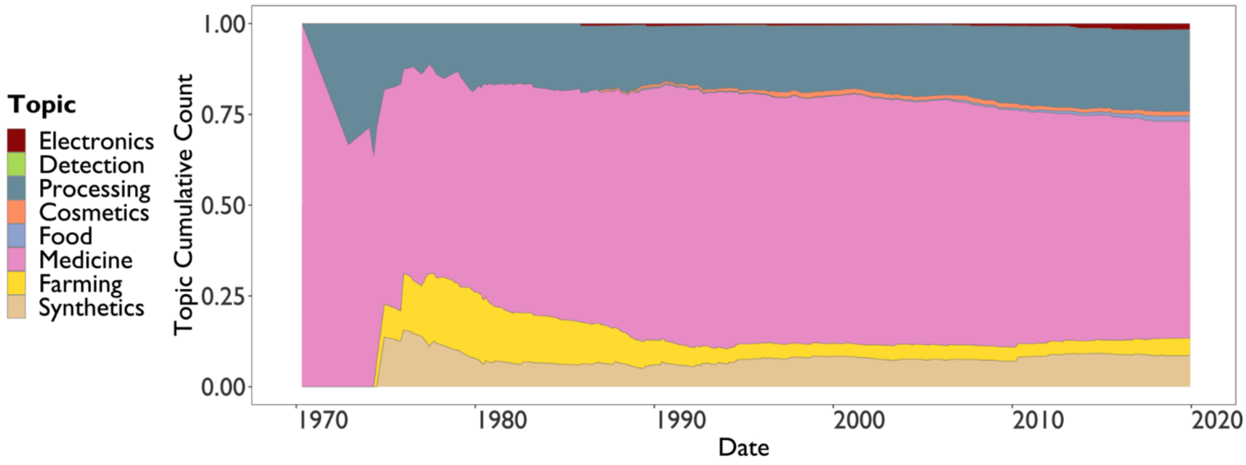

Figure S12 Manual topic model graph showing trends in topics for horseshoe crab-related patents between 1970 and 2020

Appendix 4. Manual topic keywords

Table S2. Manual keywords used to label different taxa-topic combinations in patent data scraped from the Google patent database

**Taxa**	**Topic**	**Keywords**
cordyceps	agriculture	"feed add*", "pig feed", "cow feed", "chicken feed", "duck feed", poultry, "sheep feed", "livestock feed", "fish feed", "animal feed", "feed for fowl*", "hen feed", fertili(s|z)er, fodder, pesticide, insecticid*, "animal reproduction"
pangolin	agriculture	mink, "sheep feed", "pig feed", "sow feed", "cow feed", fodder, fertili(s|z)er, swine, "dairy cow", "milk cow", "feed add*", "livestock feed", "fish feed", "animal feed", "chicken feed", "duck feed", poultry, "feed for fowl*", "hen feed", pesticide, insecticid*, fungicide
rhinoceros	agriculture	"soilless culture", fungicide, mink, "sheep feed", "pig feed", "sow feed", "cow feed", fodder, fertili(s|z)er, swine, "dairy cow", "milk cow", "feed add*", "livestock feed", "fish feed", "animal feed", "chicken feed", "duck feed", poultry, "feed for fowl*", "hen feed"
sturgeon	biotechnology	"cell line", biotech*
sturgeon	catching	catching, fishnet, fishing
cordyceps	cigarette	cigarette
sturgeon	collagen	collagen, gelatin, isinglass, chondroitin, cartilage
sturgeon	cosmetics	cosmetic*, skincare, "skin care", facial, beaut*, "face cream", "eye cream", wrinkle*, moisturi(z|s)*, toothpaste, "hair cream", shampoo, "anti aging", soap, mask, "oral hygiene", slimming, "hair growth", 化妆, 免疫, 牙膏, "skin preparation", "weight loss ", 화장품, "hair thickener"
pangolin	cosmetics	cosmetic*, skincare, "skin care", facial, beaut*, "face cream", "eye cream", wrinkle*, moisturi(z|s)*, toothpaste, "hair cream", shampoo, "anti aging", soap, mask, "oral hygiene", slimming, "hair growth", 化妆, 免疫, 牙膏, "skin preparation", "weight loss ", 화장품, "hair thickener"
bear	cosmetics	cosmetic*, skincare, "skin care", facial, beaut*, "face cream", "eye cream", wrinkle*, moisturi(z|s)*, toothpaste, "hair cream", shampoo, "anti aging", soap, mask, "oral hygiene", slimming, "hair growth", 化妆, 免疫, 牙膏, "skin preparation", "weight loss ", 화장품, "hair thickener"
horseshoe_crab	cosmetics	cosmetic*, skincare, "skin care", facial, beaut*, "face cream", "eye cream", wrinkle*, moisturi(z|s)*, toothpaste, "hair cream", shampoo, "anti aging", soap, mask, "oral hygiene", slimming, "hair growth", 化妆, 免疫, 牙膏, "skin preparation", "weight loss ", 화장품, "hair thickener"

cordyceps	cosmetics	cosmetic*, skincare, "skin care", facial, beaut*, "face cream", "eye cream", wrinkle*, moisturi(z|s)*, toothpaste, "hair cream", shampoo, "anti aging", soap, mask, "oral hygiene", slimming, "hair growth", 化妆, 免疫, 牙膏, "skin preparation", "weight loss ", 화장품, "hair thickener"
rhinoceros	cosmetics	cosmetic*, skincare, "skin care", facial, beaut*, "face cream", "eye cream", wrinkle*, moisturi(z|s)* , toothpaste, "hair cream", shampoo, "anti aging", soap, mask, "oral hygiene", slimming, "hair growth", 化妆, 免疫, 牙膏, "skin preparation", "weight loss ", 화장품, "hair thickener"
bear	detection	detect*, identification, molecular, germplasm, barcoding, differentiat*, 引物探针, fingerprint, 检测
cordyceps	detection	detect*, identification, gene, molecular, germplasm, barcoding, differentiat*, 引物探针, fingerprint, 检测
pangolin	detection	detect*, identification, gene, molecular, germplasm, barcoding, differentiat*, 引物, fingerprint, 检测
rhinoceros	detection	detect*, identification, gene, molecular, germplasm, barcoding, differentiat*, 引物, fingerprint, 检测
horseshoe_crab	detection	"species identification", fingerprint, primer (omit the word 'detection' as endotoxin detection is the main function of LAL)
sturgeon	detection	detect*, identification, barcoding, gene, genetic, molecular, germplasm, assay, differentiat*
horseshoe_crab	electronics	electrode*, capacitor, semiconductor
bear	farming	farm*, feed*, breed*, enclosure, cultivat*, drainage, "extracting bile"
pangolin	farming	enclosure, cultivat*, cage, breed*, "cub feed", "pangolin feed", "manis feed", "pangolin breeding", "breeding pangolin", "special feed", "feed for adult manis"
cordyceps	farming	farm*, cultivat*, cultur*, breed*, propagat*, larva*, inoculat*, host, incub*, grow*, planting, spawn, spore, pupa*, "fruit body"
horseshoe_crab	farming	farm, feed*, breed*, cultivat*, aquaculture, incub*, hatch*, culturing, larva*
rhinoceros	farming	farm*, feed*, breed*, enclosure, cultivat*, scraping, "artificial breeding"
sturgeon	farming	feed*, aquaculture, farm*, breed*, cultivat*, fatten*, hatch*, fishpond, pond, "parent fish", domesticat*, rear*, inseminat*, incubat*, larva*, pisciculture, reservoir, forage, "sturgeon sperm", cage, "water tank", fertili(s|z)ation, fertili(s|z)ed, reproduction, bred, pool aquaculture, fishery, broodstock, spawn*, "sturgeon fry", "medicine for sturgeon", "treating sturgeon", agriculture, "disease of sturgeon*", fishfarm*, veterinary, artificial, "sturgeon viral", "sturgeon respiratory", "sturgeon bacterial", "sturgeon food", cultur*, inspection, "fish industry", "sturgeon syringe"

pangolin	food	meat, food, coffee, tea, biscuit*, cookie*, sausage, hamburger, bacon, sauce*, soup*, wine, drink*, porridge, candy, "fruit juice", "food industry", delicious, edible, 咖啡, 茶, 饮料, 营养
sturgeon	food	caviar, "sturgeon eggs", roes?, "fish egg*", "sturgeon flesh", "fried sturgeon", sauce*, fillet*, meat, soup*, floss, canned, "food industry", "food product*", "food processing", delicious, edible, nutritious, "sturgeon extract", candy, nutritive, sausage, hamburger, bacon, wine, drink*, porridge, 咖啡, 茶, 饮料, 营养
cordyceps	food	drink*, wine, food, soup*, sauce*, biscuit*, porridge, tea, coffee, milk, "fruit juice", cake*, candy, beverage*, noodle*, delicious, nutri*, edible, sausage*, bacon, hamburger*, flavor*, taste*, 음료, 차, 맛, 饮料, 营养, 咖啡, 茶
bear	food	"bear meat", "bear paw food", coffee, tea, biscuit*, cookie*, sausage, hamburger, bacon, sauce*, soup*, wine, drink*, porridge, "fruit juice", "food industry", "food production", beverage, nutri*, 咖啡, 茶, delicious, edible, 饮料, 营养
rhinoceros	food	meat, food, coffee, tea, biscuit*, cookie*, sausage, hamburger, bacon, sauce*, soup*, wine, drink*, porridge, candy, "fruit juice", "food industry", delicious, edible, 咖啡, 茶, 饮料, 营养
horseshoe_ crab	food	meat, food
cordyceps	harvest	digging, collecting, picking, collection, excavating
sturgeon	leather	leather
bear	medicine	"for treating", "to treat", treatment, medic*, pharmac*, decoct*, health, drugs?, symptom*, sick*, pain, ointment, plasters?, pills?, therap*, syndrome, curative, detoxi*, heal*, capsule*, immun*, tablet*, wine, cardiovascular, disease*, nourishing, invigorating tonify*, disorder, patient*, illness, "dietary supplement", granule*, incense, inflamm*, injection*, cancer, tumour, tumor, eye*, liver, gallstone, hangover, "oral preparation", childbirth, *fatigue, 病, 保健, 医, 药, 酒, 治疗, 膏, 症状, 愈, 炎症
pangolin	medicine	"for treating", "to treat", treatment, medic*, pharmac*, decoct*, health, drugs?, symptom*, sick*, pain, ointment, plasters?, pills?, therap*, syndrome, curative, detoxi*, heal*, capsule*, immun*, tablet*, wine, cardiovascular, disease*, nourishing, invigorating tonify*, disorder, patient*, illness, "dietary supplement", granule*, incense, inflamm*, injection*, cancer, tumour, tumor, hangover, lactat*, nursing, "oral preparation", childbirth, *fatigue, 病, 保健, 医, 药, 酒, 治疗, 膏, 症状, 愈, 炎症
pangolin	cancer	cancer, tumour, tumor

rhinoceros	medicine	"for treating", "to treat", treatment, medic*, pharmac*, decoct*, health, drugs?, symptom*, sick*, pain, ointment, plasters?, pills?, therap*, syndrome, curative, detoxi*, heal*, capsule*, immun*, tablet*, wine, cardiovascular, disease*, nourishing, invigorating, tonify*, disorder, patient*, illness*, granule*, "dietary supplement", incense, inflamm*, injection*, cancer, tumour, tumor, "rhinoceros glue", "oral preparation", hangover, childbirth, *fatigue, 病, 保健, 医, 药, 酒, 治疗, 膏, 症状, 愈, 炎症, 약
cordyceps	medicine	"for treating", "to treat", treatment, medic*, pharmac*, decoct*, health, drugs?, symptom*, sick*, pain, ointment, plasters?, pills?, therap*, syndrome, curative, detoxi*, heal*, capsule*, immun*, tablet*, wine, cardiovascular, disease*, nourishing, invigorating, tonify*, disorder, patient*, illness*, granule*, "dietary supplement", incense, inflamm*, injection*, cancer, tumour, tumor, nursing, antibacterial, childbirth, *fatigue, peptide, "lipid lowering".病, 保健, 医, 药, 酒, 治疗, 膏, 症状, 愈, 炎症, 薬, 약, 건강, 암, 치료
horseshoe_ crab	medicine	"for treating", "to treat", treatment, medic*, pharmac*, decoct*, health, endotoxin, symptom*, curative, drugs?, biosensor, therap*, detoxi*, nourishing, invigorating, tonify*, immun*, endotoxin*, microb*, patient, illness, "dietary supplement", amebocyte, amoebocyte, amebosite, inflamm*, diagnos*, disease, biomedic*, fever, cancer, tumour, tumor, detect*, antibacterial, pyrogen*, lipopolysaccharide*, hemocyte, tachyplesin, polypeptide*, "gram negative", "gram positive", "limulus reagent", "limulus test", Perivitelline, toxin*, lectin*, childbirth, *fatigue, 病, 保健, 医, 药, 酒, 治疗, 膏, 症状, 愈, 炎症
sturgeon	medicine	"pseudosciaenae seu Acipenser", "chinese medic*", "western medic", "fish glue", "swimming bladder", plasters?, decoct*, therap*, curative, ointment, symptom*, tablets?, "air bladder", "swim bladder", maw, tea, wine, beverage, nourishing, invigorating, tonify*, pharmac*, patient, "health care", "dietary supplement", capsules?, "protein peptide*", disorder*, childbirth, fatigue, 保健
cordyceps	processing	extract*, processing, packag*, purifi*, drying, "store method", storage, bottle, cleaning, packing, crushing, grinding, powdering, cutter, cutting, box, bag, wrapping, "Production system", preserv*, separati*, concentrate, filtration, device, equipment, Encapsulation
pangolin	processing	processing, "production method"
bear	processing	extract*, processing, packaging, purifi*, "drawing bile"
sturgeon	processing	extract*, processing, packaging, purifi*, transport*, storing, extracti*, drying, freez*, filter, filtr*, preserv*
horseshoe_ crab	processing	extract*, measuring, apparatus, filtration, filter, device*, manufacturing, container, platform
sturgeon	research_co nservation	tagging, marking, monitoring, "fish ladder", sampling, , ecology, conservation, behavior, ichthyology, habitat
pangolin	research_co nservation	research, sampling, tagging, monitoring, marking, tracking, ecology, conservation, behavior

rhinoceros	snuff	snuff, tobacco
bear	synthetics	artificial*, synthe*, biotransform*, bio-transform*, bioengineer*, culturing, "producing ursodeoxycholic acid", "preparing ursodeoxycholic acid", bioconversion, bioengineered, recombinant, "preparing high purity ursodeoxycholic", 人工熊胆, "cholic acid", chenodeoxycholic, "fowl bile", 合成
pangolin	synthetics	artificial*, synthe*, biotransform*, bio-transform*, bioengineer*, cultured, substitute, bioengineered, recombinant, 合成
rhinoceros	synthetics	artificial*, synthe*, biotransform*, bio-transform*, bioengineer*, cultured, substitute, bioengineered, recombinant, 合成
cordyceps	synthetics	synthe*, biotransform*, bio-transform*, bioengineer*, recombinant, ferment*, 合成
horseshoe_crab	synthetics	rfc, rCrFC, synthe*, recombinant, biotransform*, bio-transform*, bioengineer*, "genetic engineer*", clone*, 合成
sturgeon	synthetics	synthe*, biotransformed, bio-transformed, cultured, bioengineered, recombinant, transgenic, 合成
