# Supplementary material for: Early warning of trends in commercial wildlife trade through novel machine-learning analysis of patent filing
Source: Nat Commun. 2024 Aug 1;15:6379. doi: 10.1038/s41467-024-49688-x (PMC11294592; doi:10.1038/s41467-024-49688-x)
Supplement: Supplementary file 3 — Reporting Summary [file 41467_2024_49688_MOESM3_ESM.pdf]

nature research

Corresponding author(s): Amy Hinsley
Last updated by author(s): Jun 13, 2024

Reporting Summary

Nature Research wishes to improve the reproducibility of the work that we publish. This form provides structure for consistency and transparency in reporting. For further information on Nature Research policies, see Authors & Referees and the Editorial Policy Checklist.

Statistics

For all statistical analyses, confirm that the following items are present in the figure legend, table legend, main text, or Methods section.

n/a	Confirmed	
☐	☒	The exact sample size (*n*) for each experimental group/condition, given as a discrete number and unit of measurement
☒	☐	A statement on whether measurements were taken from distinct samples or whether the same sample was measured repeatedly
☐	☒	The statistical test(s) used AND whether they are one- or two-sided<br>*Only common tests should be described solely by name; describe more complex techniques in the Methods section.*
☒	☐	A description of all covariates tested
☐	☒	A description of any assumptions or corrections, such as tests of normality and adjustment for multiple comparisons
☐	☒	A full description of the statistical parameters including central tendency (e.g. means) or other basic estimates (e.g. regression coefficient) AND variation (e.g. standard deviation) or associated estimates of uncertainty (e.g. confidence intervals)
☒	☐	For null hypothesis testing, the test statistic (e.g. *F*, *t*, *r*) with confidence intervals, effect sizes, degrees of freedom and *P* value noted<br>*Give P values as exact values whenever suitable.*
☐	☒	For Bayesian analysis, information on the choice of priors and Markov chain Monte Carlo settings
☒	☐	For hierarchical and complex designs, identification of the appropriate level for tests and full reporting of outcomes
☒	☐	Estimates of effect sizes (e.g. Cohen's *d*, Pearson's *r*), indicating how they were calculated

Our web collection on statistics for biologists contains articles on many of the points above.

Software and code

Policy information about availability of computer code

Data collection	https://ora.ox.ac.uk/objects/uuid:964753b1-d52c-4b31-8c3e-4f9c41f61004
Data analysis	https://ora.ox.ac.uk/objects/uuid:964753b1-d52c-4b31-8c3e-4f9c41f61004

For manuscripts utilizing custom algorithms or software that are central to the research but not yet described in published literature, software must be made available to editors/reviewers. We strongly encourage code deposition in a community repository (e.g. GitHub). See the Nature Research guidelines for submitting code & software for further information.

Data

Policy information about availability of data

All manuscripts must include a data availability statement. This statement should provide the following information, where applicable:

- Accession codes, unique identifiers, or web links for publicly available datasets
- A list of figures that have associated raw data
- A description of any restrictions on data availability

Data is publicly available as published patent documents, retrieved from Google Patents. As copyrighted text, however, it cannot be directly included in the code repository. All information to retrieve the dataset is available in the code, however, which is openly available at the above URL. The exact set of patents used in this study can be requested from the authors.

Field-specific reporting

Please select the one below that is the best fit for your research. If you are not sure, read the appropriate sections before making your selection.

☐ Life sciences ☒ Behavioural & social sciences ☐ Ecological, evolutionary & environmental sciences

For a reference copy of the document with all sections, see nature.com/documents/nr-reporting-summary-flat.pdf

Behavioural & social sciences study design

All studies must disclose on these points even when the disclosure is negative.

Study description	We collected quantitative data of patent counts and patenting rate from the Google Patents Database (we did not have any study participants)
Research sample	We used a dataset of published patents sourced from Google patents database
Sampling strategy	We did not sample the patents as the sampling procedure was systematic: we downloaded every patent that matched our criteria.
Data collection	Data collection procedure was systematic data scraping from the Google Patents database.
Timing	We collected data in late 2021-2022 but we focused only on historical data covering a period from 1970 to 2020, as later periods might not yet have all patents represented in the database.
Data exclusions	We did not exclude any data.
Non-participation	We did not have any participants as this was an online study of a patents database
Randomization	We did not have any participants as this was an online study of a patents database

Reporting for specific materials, systems and methods

We require information from authors about some types of materials, experimental systems and methods used in many studies. Here, indicate whether each material, system or method listed is relevant to your study. If you are not sure if a list item applies to your research, read the appropriate section before selecting a response.

Materials & experimental systems

n/a	Involved in the study
☒	☐ Antibodies
☒	☐ Eukaryotic cell lines
☒	☐ Palaeontology
☒	☐ Animals and other organisms
☒	☐ Human research participants
☒	☐ Clinical data

Methods

n/a	Involved in the study
☒	☐ ChIP-seq
☒	☐ Flow cytometry
☒	☐ MRI-based neuroimaging
